# Ketodex: A Game-Changer in Pediatric Sedation for Challenging Airway

**DOI:** 10.7759/cureus.75959

**Published:** 2024-12-18

**Authors:** Lidia Sofia Baptista, Jorge Pelicano Paulos, Ana Pinto Carneiro

**Affiliations:** 1 Anesthesiology, Unidade Local de Saúde (ULS) de Loures-Odivelas, Hospital Beatriz Ângelo, Loures, PRT; 2 Anesthesiology, Unidade Local de Saúde (ULS) de São José, Hospital Dona Estefânia, Lisbon, PRT

**Keywords:** dexmedetomidine, difficult airway, ketamine, pediatric anesthesia, safety, sedation

## Abstract

Managing sedation in pediatric patients with complex facial anomalies and airway challenges requires careful consideration of safety and efficacy. This case report presents the use of the Ketodex sedation protocol, combining ketamine (NMDA receptor antagonist) and dexmedetomidine (alpha-2-agonist), for a child with a large cervical/facial mass undergoing a diagnostic magnetic resonance imaging (MRI).

Ketodex provides effective sedation with minimal need for manipulation of the airway and side effects, making it ideal for cases involving difficult airways. Our protocol allows spontaneous ventilation without compromising airway reflexes and reduces airway hyperreactivity, ensuring safety in high-risk pediatric cases. Initiating sedation in a controlled environment enhances safety before moving to remote locations.

The Ketodex protocol is a safe and effective choice for managing pediatric patients with challenging airways. Its use should be prioritized in controlled settings, with adaptations for individual patient needs.

## Introduction

Hospital Dona Estefânia, the largest pediatric hospital in Portugal, performs numerous imaging exams daily with anesthetic support. Most procedures are carried out under inhaled sedation with sevoflurane through a facial mask. However, for patients with facial dysmorphia or complex airway anomalies, the standard anesthetic approach can be risky, requiring adjustments to ensure safety and effectiveness [1]. This case report discusses the use of the Ketodex sedation protocol for a pediatric patient with a large facial mass and subsequent predictable difficult airway, highlighting its advantages in maintaining spontaneous ventilation and minimizing airway manipulation risks.

## Case presentation

A four-year-old male patient, weighing 17 kg and classified as American Society of Anesthesiologists (ASA) physical status III, was evacuated from Guinea-Bissau due to a large congenital cervical/facial mass, requiring specialized surgical care. This mass, present since birth, had progressively enlarged, causing intermittent episodes of mild hemorrhage and swallowing difficulties.

**Figure 1:**
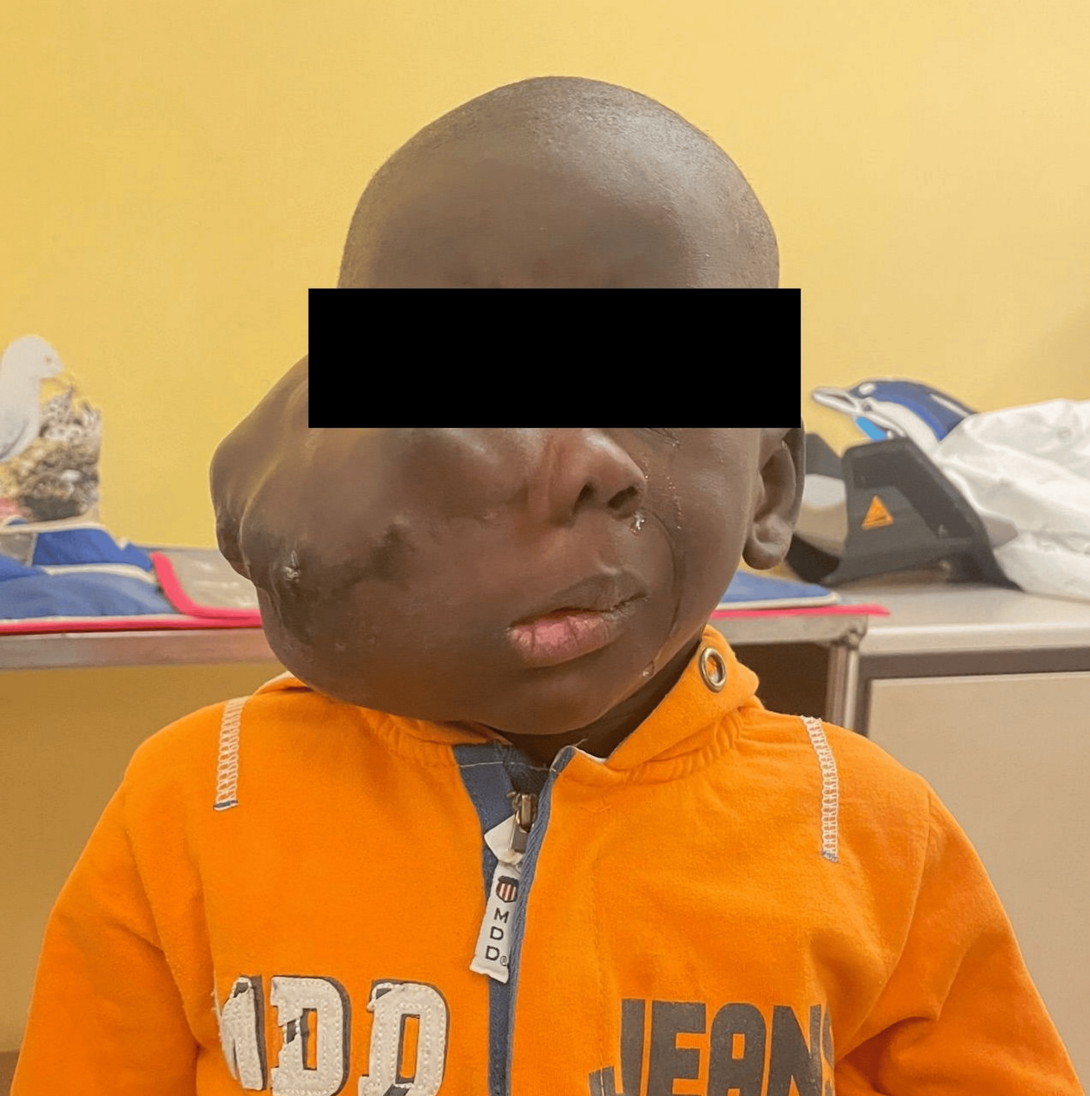
Four-year-old male with a congenital cervical/facial vascular malformation.

On examination, a large mass was located in the right hemiface, extending into the cervical region (Figure 1). It caused superior orbital deviation, left septum displacement, and nasal cavity compression. A head and neck MRI was proposed to clarify the nature of the lesion, suggesting it to be a vascular malformation. This patient had never been sedated or anesthetized before. 

Given the complexity of his airway approach, which precluded the use of a facial mask, a Ketodex sedation protocol was chosen. The protocol included an induction bolus of ketamine (1 mg/kg) and dexmedetomidine (1 mcg/kg), followed by a continuous infusion of ketamine (1 mg/mL) and dexmedetomidine (1 mcg/mL) at 0.2-2 mL/kg/h, adjusted according to hemodynamic and neurological monitoring. 

Sedation was initiated in the operating room (OR) to ensure access to advanced airway management eventually needed tools, including a video laryngoscope, bronchoscope, and other airway adjuvants. After the bolus, the infusion rate was set at 0.4 mL/kg/h (of the mentioned Ketodex dilution). Once the patient was adequately sedated, he was transferred to the imaging department, where the MRI was successfully performed without complications, reaching a maximum infusion rate of 0.7 mL/kg/h. During transport and the imaging exam, the patient was continuously monitored. No changes in heart rate or blood pressure were observed. 

If sedation with Ketodex had not been effective, given that the use of a face mask was not feasible, and this would undoubtedly be a difficult airway, plan B would be the use of advanced airway management tools, awake fiberoptic intubation, or, as a last resort, a surgical airway. 

## Discussion

Unlike adults, children are more anxious and uncooperative in clinical procedures. Even in non-painful procedures, it may be necessary for children to remain still, which is why sedation strategies are required [2]. 

In pediatric imaging procedures requiring sedation, inhalational anesthesia is often chosen due to its non-invasive administration and rapid recovery profile. Agents like sevoflurane are preferably used in our center, offering effective sedation with minimal impact on airway protective reflexes, which is particularly beneficial for young children or those who may have difficulty cooperating during procedures [3,4].

Regarding intravenous sedation strategies, while propofol and midazolam are commonly employed for sedation in pediatric patients, they may correlate with a higher incidence of respiratory complications, such as airway obstruction and hypoventilation, which may necessitate airway interventions [5].

On the other hand, the combination of ketamine and dexmedetomidine (Ketodex) offers unique advantages for pediatric sedation, particularly in cases involving difficult airways. Ketamine provides dissociative anesthesia, ensuring the maintenance of spontaneous ventilation and airway reflexes, while dexmedetomidine balances its effects while inducing sedation and analgesia with minimal respiratory depression. Together, these drugs create a synergistic effect that enhances safety in high-risk scenarios.

Pediatric patients with complex airway anatomy, as in this case report, pose significant challenges for anesthetic management. Ketamine's bronchodilating properties and its ability to maintain respiratory drive make it a crucial agent in such difficult airway cases [6,7]. Dexmedetomidine complements this by providing a stable sedative state with minimal hemodynamic changes, reducing airway hyperreactivity and stress responses [8,9].

Studies have demonstrated that the Ketodex protocol is effective and safe for these high-risk pediatric patients [10]. Tavares et al. highlighted its efficacy in low-resource settings, emphasizing its adaptability and safety profile [6]. Its growing use has also been described in cases of craniofacial anomalies, where standard sedation methods may not be viable [9].

The most frequent complications are bradycardia, mild hypotension, hypersecretion, and prolonged sedation. However, these are manageable with proper monitoring and dose adjustments. In this case, initiating sedation in the OR provided a controlled environment with immediate access to difficult airway management tools, a strategy recommended in similar situations to mitigate risks [11,12].

## Conclusions

The management of difficult airway in pediatric patients requires careful planning and the use of sedation protocols that prioritize spontaneous ventilation and airway protection. Ketodex is a reliable option in these scenarios, offering sedation stability with minimal respiratory compromise. Its application in controlled environments, such as the operating room, ensures enhanced safety, particularly for high-risk patients with complex anatomical challenges. The ability to adapt protocols to individual patient needs is crucial for successful outcomes in pediatric anesthesia.
